# Myeloid dendritic cells stimulated by thymic stromal lymphopoietin promote Th2 immune responses and the pathogenesis of oral lichen planus

**DOI:** 10.1371/journal.pone.0173017

**Published:** 2017-03-09

**Authors:** Masaki Yamauchi, Masafumi Moriyama, Jun-Nosuke Hayashida, Takashi Maehara, Noriko Ishiguro, Keigo Kubota, Sachiko Furukawa, Miho Ohta, Mizuki Sakamoto, Akihiko Tanaka, Seiji Nakamura

**Affiliations:** 1 Section of Oral and Maxillofacial Oncology, Division of Maxillofacial Diagnostic and Surgical Sciences, Faculty of Dental Science, Kyushu University, Fukuoka, Japan; 2 OBT Research Center, Faculty of Dental Science, Kyushu University, Fukuoka, Japan; Toho Daigaku, JAPAN

## Abstract

Oral lichen planus (OLP) is a chronic inflammatory disease characterized by subepithelial T-cell infiltration. Recent studies reported that specific T helper (Th) subsets, especially Th2 cells, are involved in the pathogenesis of OLP. Thymic stromal lymphopoietin (TSLP) is mainly secreted by epithelial cells and potently activates myeloid dendritic cells (mDCs) to induce Th2-mediated inflammation. Here, we investigated the expression of TSLP and related molecules in OLP. Buccal mucosa specimens from patients with OLP, hyperkeratosis, and ulcer were analyzed by immunohistochemistry for expression of TSLP, its receptor (TSLPR), and inflammatory cells. TSLP was detected in/around the epithelium of patients with OLP and hyperkeratosis, whereas TSLPR, CD11c (mDC), and GATA3 (Th2) were strongly expressed in the subepithelial layer only in OLP patients. Double immunofluorescence staining showed that TSLPR expression mainly co-localized with CD11c. Moreover, the number of CD11c- and GATA-3 positive cells was correlated in OLP patients. In lesions selectively extracted by laser microdissection, the mRNA expression of Th2 (IL-4, MDC, TARC, GATA3)- and Th17 (IL-17, RORγt)-related molecules in OLP patients was significantly higher than in other groups. These results suggest that CD11c^+^ mDCs expressing TSLPR contribute to aberrant Th2 immune responses and the pathogenesis of OLP via TSLP stimulation.

## Introduction

Oral lichen planus (OLP) is a chronic inflammatory disease characterized by abnormally keratinized oral mucosa and band-like T-cell infiltration in the upper lamina propria. Consequently, it is referred to as a potentially malignant disorder by the World Health Organization working group [1–3]. Clinically, OLP is classified into seven forms: atrophic, bullous, erosive, popular, pigmented, plaquelike; or reticular. The patients with reticular lesions, the most common form, generally have no clinical symptoms, while atrophic, bullous, and erosive lesions cause pain, ranging from mild to severe. Notably, erosive OLP shows a significantly higher rate of malignant transformation than non-erosive OLP [3].

Immunologically, CD4^+^ T helper (Th) cells, including Th1, Th2, Treg, and Th17 subsets, are thought to play an important role in the pathogenesis of OLP, via their specific cytokine networks [4–6]. Recently, Liu *et al*. [7, 8] found that levels of the Th2 cytokine, IL-4, in the serum and saliva of OLP patients were significantly higher than those in healthy controls, and that the salivary IL-4 level in the erythematous/ulcerative group was significantly higher than in the reticular group. These data indicate the possibility of salivary IL-4 as a biomarker for monitoring the diagnosis and severity of OLP, but also suggest a Th2-predominant immune imbalance in OLP.

However, to our knowledge, no published reports have investigated the mechanism of Th2-cell activation in OLP. Thymic stromal lymphopoietin (TSLP), a cytokine belonging to the IL-2 family, has recently been investigated as a Th2-inducing cytokine [9]. It is produced by epithelial cells, mast cells, and fibroblasts. In bronchial asthma and atopic dermatitis, TSLP activated myeloid dendritic cells (mDC) and monocytes through its receptor (TSLPR) to abundantly produce Th2-related chemokines including MDC and TARC, resulting in migration of Th2 cells to the lesions [10, 11]. In this study, we thus examined the expression of TSLP and the distribution of infiltrating TSLPR-expressing cells in lesions of the buccal mucosa (BM) to clarify the contribution of TSLP/TSLPR signaling to the pathogenesis of OLP.

## Materials and methods

### Ethics statement

This study design was approved by the Ethics Committee of Kyushu University, Japan, and written informed consent was obtained from all of the patients and healthy controls (IRB serial number: 25–287).

### Patients

BM samples were collected from patients referred to the Department of Oral and Maxillofacial Surgery, Kyushu University Hospital between 1997 and 2015. Forty-three patients with OLP (13 men and 30 women; mean age, 64.0 ± 11.1 years), 5 patients with hyperkeratosis (2 men and 3 women; mean age, 42 ± 4.1 years), and 5 patients with ulcer (3 men and 2 women; mean age, 36.4 ± 5.6 years) participated in this study. The diagnosis of each disease was made by clinical features and histopathological findings. The clinical characteristics of the 43 patients with OLP are summarized in Table 1.

**Table 1:** Association of Th2-related molecules with clinical characteristics in 43 patients with OLP.

Case (%)		CD11c^+^ cells (/HPF)	*P*-value	GATA3^+^ cells (/HPF)	*P*-value
**Age**†					
≤ 65	24 (55.8)	88.7 ± 63.6		154.3 ± 115.3	
65 <	19 (54.2)	99.3 ± 154.3	*N*.*S*.	187.8 ± 163.3	*N*.*S*.
**Gender**†					
Male	13 (30.2)	92.6 ± 78.8		165.8 ± 143.7	
Female	30 (69.8)	93.8 ± 65.4	*N*.*S*.	170.6 ± 137.8	*N*.*S*.
**Affected site**†					
Tongue	4 (9.3)	64.5 ± 88.1		74.4 ± 94.2	
Gingiva	11 (25.6)	69.9 ± 73.1		139.7 ± 159.8	
Buccal mucosa	28 (65.1)	106.8 ± 61.2	*N*.*S*.	194.2 ± 127.9	*N*.*S*.
**Clinical form** †					
Reticular type	20 (46.5)	68.9 ± 62.4		114.4 ± 108.9	
Erosive type	23 (53.5)	114.7 ± 68.8	0.0243	216.8 ± 145.7	0.0374
**Viral infection**†					
**+**	6 (14.0)	114.1 ± 75.8		202.3 ± 134.8	
**−**	37 (86.0)	94.6 ± 72.8	*N*.*S*.	171.4 ± 145.3	*N*.*S*.
**eGC formation**†					
**+**	5 (11.6)	163.3 ± 80.0		331.6 ± 138.4	
**−**	38 (88.4)	84.2 ± 62.7	0.0488	147.8 ± 124.9	0.0131
**Resistance of steroid therap**†					
**+**	7 (16.3)	77.1 ± 28.8		155.4 ± 107.5	
**−**	36 (83.7)	96.6 ± 74.7	*N*.*S*.	171.8 ± 144.9	*N*.*S*.
**Canceration**†					
**+**	4 (9.3)	47.5 ± 17.0		90.4 ± 89.3	
**−**	39 (90.7)	98.1 ± 71.4	*N*.*S*.	177.2 ± 141.3	*N*.*S*.

Th2, T helper type 2; OLP, oral lichen planus; HPF, high-power field; eGC, ectopic germinal center; *N*.*S*., not significant.

^†^ Mann-Whitney *U*-test and Wilcoxon signed-rank test.

### Immunohistochemistry

Four-μm formalin-fixed and paraffin-embedded (FFPE) sections of specimens from patients with OLP, ulcer and hyperkeratosis were prepared and stained with a conventional avidin—biotin complex technique as previously described [12]. The mouse monoclonal antibodies used to analyze the protein expression were anti-CD11c (ab212508; Abcam, Cambridge, MA, USA), and anti-CD68 (ab995; Abcam). Anti-GATA3 (ab199428; Abcam) was a rabbit monoclonal antibody used to analyze the Th2 transcription factor, GATA-3. Rabbit polyclonal antibodies: anti-IFN-γ (500-P32; Peprotech, Rocky Hill, NJ, USA), anti-TSLP (ab47943; Abcam), anti-CRLF2 (ab109626; Abcam), and anti-IL-17 (13082-1-AP; Proteintech, Chicago, IL) were used. Tissue sections were sequentially incubated with primary antibodies for 2.5 h, then with biotinylated anti-mouse IgG or anti-rabbit IgG secondary antibodies (Vector Laboratories, Burlingame, CA, US), avidin—biotin—horseradish peroxidase complex (Vector Laboratories), and 3,3′-diaminobenzidine (Vector Laboratories). Mayer's hematoxylin was used for counterstaining. Photomicrographs were obtained using a light microscope equipped with a digital camera (BZ-9000 series; Keyence, Tokyo, Japan).

### Double immunofluorescence analysis

For double immunofluorescence analysis, 4 μm FFPE sections were prepared and stained. Sections were incubated with the primary antibody, anti-CD11c (Abcam), or anti-CD68 (Abcam), at room temperature for 2 h after blocking with 1% BSA for 1 h, then incubated with secondary antibody (1:400 dilution of anti-mouse IgG conjugated with Alexa 488 USA) for 30 min. The sections were washed, blocked with 1% BSA blocking buffer for 40 min, and incubated with the primary antibody, anti-CRLF2 (Abcam) at room temperature for 2 h. After incubation, the sections were incubated with secondary antibodies (1:400 dilution of anti-rabbit IgG conjugated with Alexa 546) for 30 min at room temperature. Slides were mounted (VectaMount, Vector Laboratories) and kept in the dark. DAPI was used to stain nuclei. Images were taken using a Keyence microscope (BZ-9000 series), setting the background fluorescence level with negative controls.

### Evaluation of TSLP, TSLP-related molecules positive cells

The number of TSLP, TSLPR, CD11c, and GATA3 positive cells in immunohistochemical staining was counted in 4 mm^2^ sections from five independent high-power microscopic fields (400×; 0.0625 μm^2^) in the subepithelial layer. In the same way, the number of CD11c^+^TSLPR^+^ and CD68^+^TSLPR^+^ cells in double immunofluorescence staining was counted.

### Tissue sampling by LMD

LMD was performed using a Leica Microdissection system (LMD6500; LeicaMicrosystems Japan, Tokyo, Japan), as described previously [13–15]. In preparation for LMD, 20 μm-thick FFPE sections were cut from OLP, ulcer and hyperkeratosis specimens. After deparaffinizing and hydrating, sections were washed in distilled water. Specimens were stained with hematoxylin for 5 min and eosin for 1 min, washed in distilled water, and air-dried with a fan for 30 s. The sections were treated with xylene for 3 min. All reagents were prepared RNase-free, and the entire process was performed quickly to minimize RNA degradation. Briefly, the tissue area of interest was positioned and cut out using a focused pulsed laser beam. Dissected areas were collected in the cap of a microcentrifuge tube via laser pressure catapulting. The cap was filled with 65 μl Buffer PKD (RNeasy FFPE Kit, Qiagen). To obtain enough RNA for a stable real-time quantitative polymerase chain reaction (PCR), at least 20 FFPE sections for each patient were collected, as recommended, depending on cell density. mRNA expression for all experiments was confirmed by PCR (data not shown).

### RNA extraction from microdissected samples and cDNA synthesis

Total RNA was extracted independently from the LMD samples using the RNeasy FFPE Kit (Qiagen) as per manufacturer’s instructions and as described previously [15]. Total RNA samples (1 μg) were prepared and used for the synthesis of cDNA and then RNA was incubated for 1 hour at 42°C with 20 U of RNase inhibitor (Promega Corp.), 0.5 mg of oligo-1218 (Pharmacia, Uppsala, Sweden), 0.5 mM of each deoxyribonucleotide triphosphate (dNTP) (Pharmacia), 10mM of dithiothreitol (DTT), and 100 U of RNA reverse transcriptase (Life Technologies, Gaithersburg, MD).

### Quantitative estimation of mRNA by real-time PCR

The resulting cDNA was amplified using Light Cycler Fast Start DNA Master mix SYBR Green III (Roche Diagnostics, Mannheim, Germany) in a Light Cycler real-time PCR instrument (version 3.5; Roche Diagnostics). The mRNA levels of GATA3, MDC, TARC, IL-4, TSLP, TSLPR, CD11c, CD123, T-bet, IFN-γ, IL-17, and RORγt were analyzed. The primer sequences used were as follows: GATA3 (150bp), forward 5’-GGA GGT GGA TGT GCT TTT TA -3’, reverse 5’-GGT AGG GAT CCA TGA AGC A -3’; MDC (227bp), forward 5’-CCT ACA GAC TGC ACT CCT GGT T -3’, reverse 5’-GAT CGG CAC AGA TCT CCT TAT C -3’; TARC (140bp), forward 5’-TAG AAA GCT GAA GAC GTG GT -3’, reverse 5’-GGC TTT GCA GGT ATT TAA CT -3’; IL-4 (90bp), forward 5’-AGC TGA TCC GAT TCC TGA AAC -3’, reverse 5’-TAC TCT GGT TGG CTT CCT TCA C -3’; TSLP (117bp), forward 5’-CCC AGG CTA TTC GGA AAC TC -3’, reverse 5’-CGC CAC AAT CCT TGT AAT TG -3’; TSLPR (166bp), forward 5’-TGA CGT GTT CTG ACC TGT CC -3’, reverse 5’-TCC ATA GCC TTC ACC CTG AC -3’; CD11c (215bp), forward 5’-AGG ACA TTG TGT TCC TGA TCG -3’, reverse 5’-CTG GTG AAC AGA AGC CAA CAG -3’; CD123 (216bp), forward 5’-TCC CCT GCA CAG ATA AGT TTG -3’, reverse 5’-CGT TCC AGG ATT GAG TAG CTG -3’; T-bet (310bp), forward 5’-GCA AGA CCT GTA CGC CAA C -3’, reverse 5’- ATC TCC CCC AAG GAA TTG AC -3’; IFN-γ (142bp), forward 5’-TGT CCA ACG CAA AGC AAT AC -3’, reverse 5’-ATA TTG CAG GCA GGA CAA CC -3’; IL-17 (189bp), forward 5’-GAC TCC TGG GAA GAC CTC ATT -3’, reverse 5’-GAG GAC CTT TTG GGA TTG GTA -3’; RORγt (211bp), forward 5’-GGG TAC AAT GAA GGC CAA GA -3’, reverse 5’-AGC TGT GGC CTC AAG GAT AA -3’;and β-actin (258bp), forward 5’-GCA AGA CCT GTA CGC CAA C -3’, reverse 5’-CTA GAA GCA TTT GCG GTG GA -3’. The relative mRNA levels were calculated after normalizing to the housekeeping gene β-actin.

### Statistical analysis

All statistical analyses in the present study were performed using JMP software version 13 (SAS Institute, NC, USA). The significance of differences between groups was determined using Mann—Whitney *U* tests, Kruskal—Wallis tests, and Spearman rank correlations. A *P*-value of *P* < 0.05 was considered statistically significant.

## Results

### Expression of TSLP and TSLPR in BM

**Fig 1:**
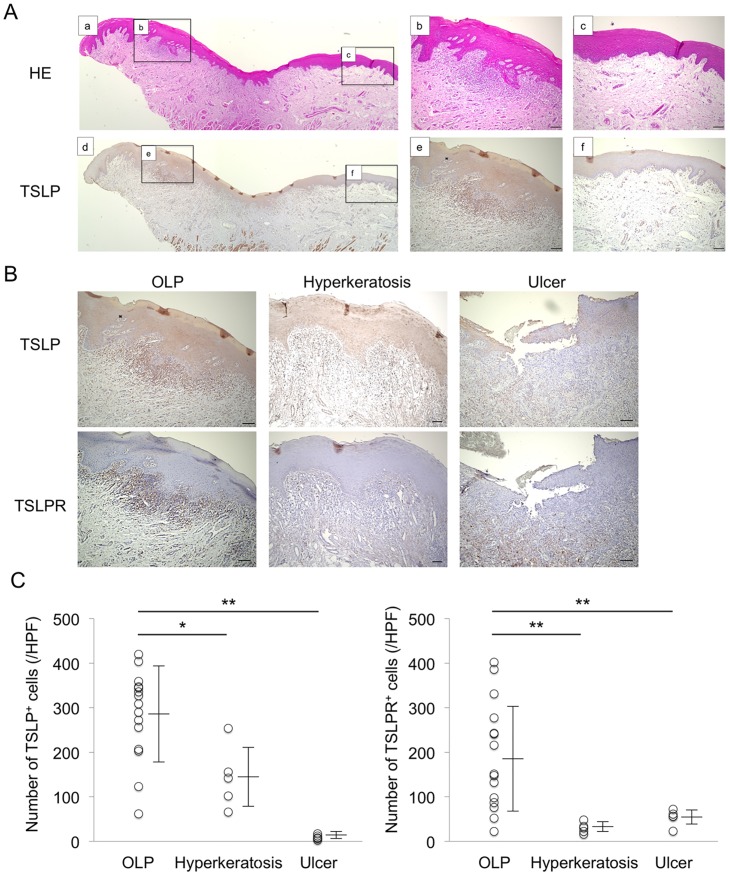
Localization of Thymic Stromal Lymphopoietin (TSLP) in Buccal Mucosa (BM) specimens from patients with Oral Lichen Planus (OLP). **A**, Representative images of paraffin sections stained with hematoxylin and eosin (a-c) and TSLP antibodies (brown) (d-f). Comparative examination was performed between lesion (b, e) and normal section (c, f). Counterstaining with Mayer’s hematoxylin was subsequently performed (blue). Scale bars, 100 μm. **B**, Distribution of TSLP and TSLPR in BM specimens from representative patients with OLP, hyperkeratosis and ulcer. Counterstaining was performed with Mayer's hematoxylin (blue). Scale bars, 100 μm. **C**, The number of TSLP^+^ and TSLPR^+^ cells in BM specimens from patients with OLP (n = 15), hyperkeratosis (n = 5) and ulcer (n = 5). The number of these positive cells was calculated from immunohistochemical staining as described in the Methods section. Statistical significance of differences between groups was determined by Kruskal-Wallis tests (**P < 0.01, *P < 0.05).

BM specimens from patients with OLP, hyperkeratosis or ulcer were examined after immunohistochemical staining to evaluate the distributions of TSLP and TSLPR. As shown in Fig 1A, expression of TSLP was strongly detected in/around the epithelium in the lesions, but not in the normal tissues, of patients with OLP. Moreover, expression of TSLP was weakly detected in the epithelium from patients with hyperkeratosis, but was rarely seen in the lesions from patients with ulcer. On the other hand, expression of TSLPR was only detected in the subepithelium of patients with OLP, where it was strongly detected in infiltrating cells (Fig 1B). In addition, the number of TSLP^+^ and TSLPR^+^ cells in the subepithelium from patients with OLP was significantly higher than that from other groups (Fig 1C).

### Expression of TSLPR-expressing cells in BM

**Fig 2:**
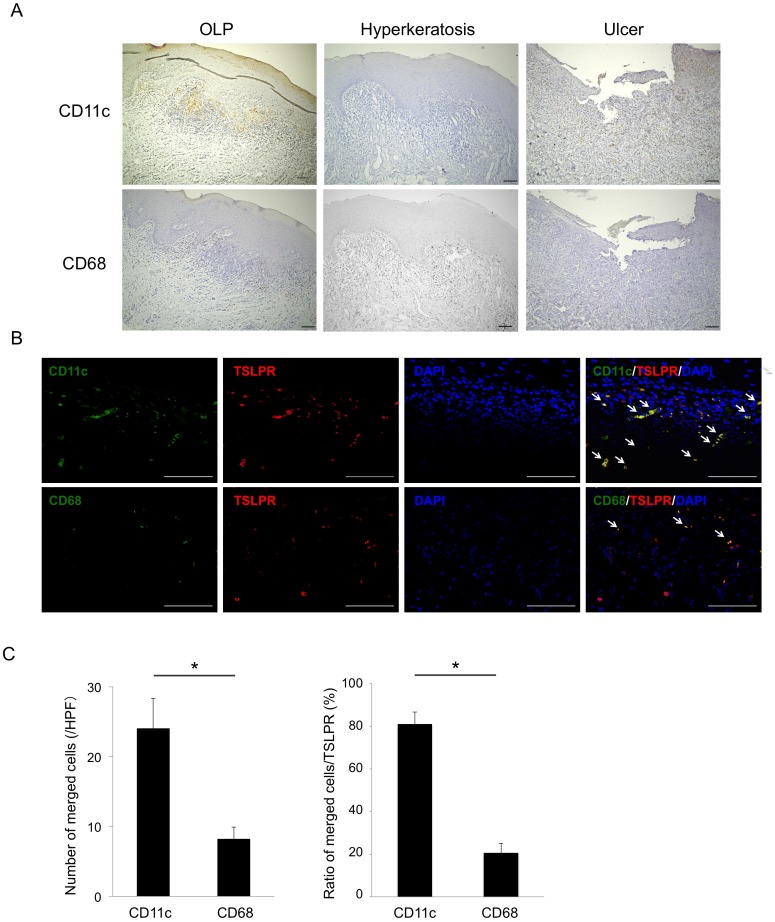
Expression and localization of TSLPR-expressing cells in the BM specimens. **A**, Representative images of paraffin sections stained with CD11c and CD68 antibodies (brown). Counterstaining with Mayer’s hematoxylin was subsequently performed (blue). Scale bars, 100 μm. **B**, Double immunofluorescence staining performed with anti-CD11c (green) or anti-CD68 (green), anti-TSLPR (red), and DAPI for staining nuclei (blue). White arrows indicate co-localizing staining of cells (yellow). Scale bars, 100 μm. **C**, The number and ratio of merged cells to TSLPR^+^ cells from 15 patients with OLP calculated by double immunofluorescence staining. Statistical significance of differences between groups was determined by Mann—Whitney *U* tests (*P < 0.05).

As TSLPR is known to be expressed on mDCs (CD11c^+^) and monocytes (CD68^+^), we next compared the expression and distribution of TSLPR-expressing cells among each group. Expression of CD68 was diffusely detected in infiltrating cells in the subepithelium from all groups. Interestingly, expression of CD11c was selectively detected in infiltrating cells beneath the basal layer, only in patients with OLP (Fig 2A), indicating that the distribution of CD11c was similar to that of TSLPR in patients with OLP.

### Co-localization of TSLPR and cell markers in BM of patients with OLP

To clarify which cells express TSLPR in patients with OLP, double immunofluorescence staining with antibodies detecting TSLPR (red) and CD11c or CD68 (green) was performed. Using this approach, we found that TSLPR co-localized with CD11c, and only partly co-localized with CD68 (Fig 2B). To confirm this, semi-quantitative analysis of CD11c^+^TSLPR^+^ and CD68^+^TSLPR^+^ cells in BM specimens by double immunofluorescence staining showed that the number and percentage of CD11c^+^TSLPR^+^ cells was higher than those of CD68^+^TSLPR^+^ cells (Fig 2C).

### Expression of Th-related cytokines, chemokines, and transcription factors in BM

**Fig 3:**
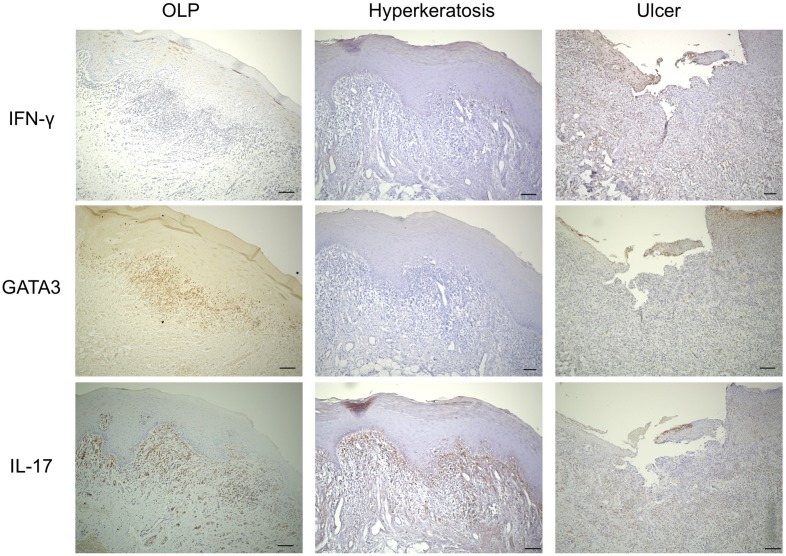
Localization of T helper (Th) subsets in the BM specimens. Representative images of paraffin sections stained with IFN-γ, GATA3, and IL-17 from patients with OLP, hyperkeratosis, and ulcer. Counterstaining with Mayer’s hematoxylin was subsequently performed (blue). Scale bars, 100 μm.

As shown in Fig 3, the specimens were examined immunohistochemically to evaluate the distribution of Th subsets in the BMs from each group. Expression of IFN-γ (Th1 cytokine) was detected at low levels in cells infiltrating the subepithelium of patients with OLP and ulcer, while expression of IL-17 (Th17 cytokine) was also detected in the subepithelium of patients with OLP and hyperkeratosis. Interestingly, expression of GATA3 (Th2-related transcription factor), TSLP, TSLPR, and CD11c was strongly detected in infiltrating cells in the subepithelium only in patients with OLP.

**Fig 4:**
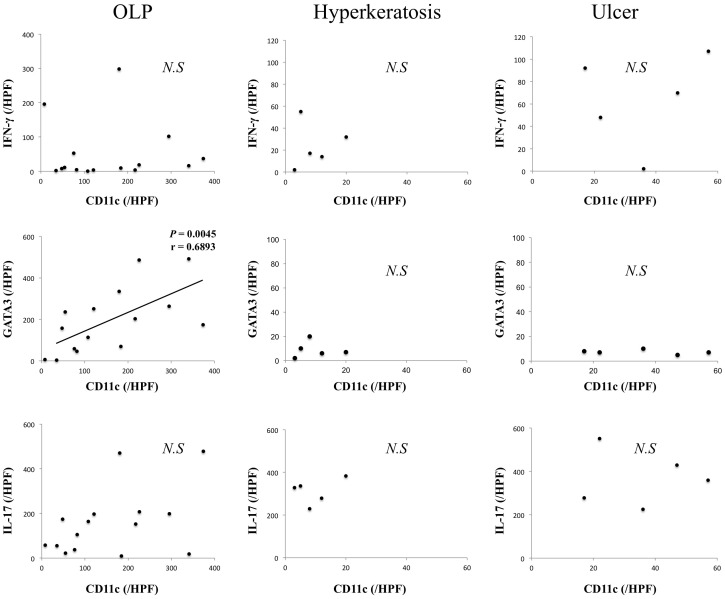
Correlation between the number of CD11c^+^ and GATA3^+^ cells in BM specimens. The number of these positive cells in BM samples from patients with oral lichen planus (OLP) (n = 15), hyperkeratosis (n = 5) and ulcer (n = 5) was calculated from immunohistochemical staining as described in the Methods section. Statistical significance of differences between groups was determined by Spearman’s rank correlation (*p* < 0.05).

Moreover, the association of mDCs with accumulation of Th2 cells in each group were examined. The number of CD11c^+^ cells was positively correlated with that of GATA3^+^ cells only in patients with OLP (Fig 4).

### Comparison of the mRNA expression levels of Th-related cytokines and transcription factors in the infiltrating cells the subepithelium

**Fig 5:**
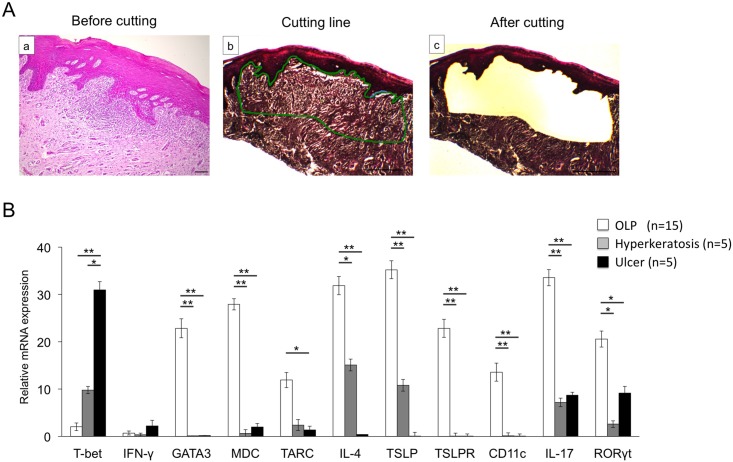
mRNA expression of Th-related cytokines, chemokines, and transcription factors in the selective BM specimens by using Laser Microdissection (LMD). **A**, Hematoxylin and eosin (HE)-stained BM specimens (a). The infiltration of inflammatory cells was indicated by a green line (b). The preselected area was extracted by using a guided laser beam after LMD. (c). Scale bars, 500 μm. **B**, mRNA expression levels of Th-related cytokines, chemokines, and transcription factors in BM samples from patients with OLP (n = 15), hyperkeratosis (n = 5) and ulcer (n = 5). mRNA expression levels of Th-related cytokines, chemokines, and transcription factors were estimated quantitatively as described in the Methods section. Statistical significance of differences between groups was determined by Kruskal-Wallis tests (**P < 0.01, *P < 0.05).

To quantitatively evaluate the mRNA expression levels of Th-related cytokines, chemokines, and transcription factors restricted to infiltrating cells, subepithelium samples of BM specimens were isolated by LMD (Fig 5A). As shown in Fig 5B, the mRNA expression of GATA3, MDC, TARC, IL-4, IL-17, and RORγt in infiltrating cells from patients with OLP was significantly higher than that from other groups. In contrast, expression of T-bet in infiltrating cells from patients with ulcer was significantly higher than that from other groups. The mRNA expression of IFN-γ showed no statistically significant difference between groups.

### Associations of mDCs and Th2 cells with clinical findings of patients with OLP

The associations of mDCs and Th2 cells with the clinical findings of the OLP patients were examined. As shown in Table 1, the number of CD11c^+^ and GATA3^+^cells in erosive type lesions or those with ectopic germinal center (eGC) formation showed significant increases compared to the number of these cells in reticular type lesions or those without eGC formation. In contrast, the number of CD11c^+^ and GATA3^+^cells was not associated with other clinical parameters such as age, gender, affected site, viral infection, resistance to steroid therapy, and canceration.

## Discussion

Several studies have reported that TSLP promotes production of Th2-related chemokines such as MDC and TARC by mDC and increases disease severity in asthma, atopic dermatitis and T-cell lymphoma [10, 16–18]. In addition, we recently examined the expression of Th-related cytokines and chemokines in the labial salivary glands and BM of patients with chronic graft versus host disease (cGVHD) characterized by subepithelial lymphocytic infiltration. These results suggest that cGVHD might be initiated and/or maintained by Th1 cells and thereafter progresses in association with Th2 cell accumulation via the interaction of particular chemokine and chemokine receptors [19]. In the present study, we found that the expression of TSLP, CD11c, GATA3, MDC, and TARC in patients with OLP was significantly higher than other groups, and were consistent with recent studies. This is the first comparative study suggesting the involvement of TSLP and Th2-related chemokines in the pathogenesis of OLP.

Soumelis *et al*. [10] reported that TSLP strongly activates CD11c^+^ mDCs expressing its cognate receptor (TSLPR) to produce Th2-related chemokines. DCs are well known to play an important role in the pathogenesis of allergic diseases and autoimmune diseases [20]. In humans, several DC subsets have been identified based on their function, including CD11c^+^CD123^-^ mDCs and CD11c^-^CD123^+^ plasmacytoid DCs (pDC) [21, 22]. mDCs promote Th1 development and cytotoxic T-cell responses by production of IL-12. In contrast, pDCs provide a milieu favoring Th2-biased immune reactions by production of IFN-α. Interestingly, mDCs express high levels of TSLPR and produce Th2-related chemokines, thus priming Th2 cell development after stimulation with TSLP [10]. In OLP, we found that CD123^+^ pDCs were rarely seen in the subepithelium of BM specimens (S1 Fig), whereas CD11c^+^ mDCs were observed as infiltrating cells beneath the basal layer, and the number of CD11c^+^ mDCs positively correlated with the number of GATA3^+^ Th2 cells. In addition, the number of CD11c^+^ mDCs and GATA3^+^ Th2 cells associated with clinical findings including the degree of mucosal inflammation and the presence of eGC formation.

In conclusion, we have demonstrated that CD11c^+^TSLPR^+^ mDCs promote the migration of Th2 cells and increase the severity of OLP by TSLP production from the lesional epithelium. Zhang *et al*. [23] indicated that TSLP also directly induced Th2 differentiation of naive CD4^+^ Th cells, and activated natural killer T cells and basophils at the early stage of inflammation. Additional investigation is required to determine the direct effects on Th2 cells of TSLP stimulation and the role of Th2 cells attracted by mDCs in the OLP mucosa. Since OLP is sometimes resistant to steroid therapy as a first-line therapy, a more thorough understanding of the function of Th2 cells could lead to the development of novel pharmacological strategies aimed at disrupting Th2 cells or their activators, and inhibiting the initiation or progression of OLP.

## Supporting information

S1 Fig Expression and localization of CD123 and TSLPR-expressing cells in the BM specimens. **A**, Representative images of paraffin sections stained with CD123 and TSLPR antibodies (brown). Counterstaining with Mayer’s hematoxylin was subsequently performed (blue). Scale bars, 100 μm. **B**, mRNA expression levels of CD123 in BM samples from patients with OLP (n = 15), hyperkeratosis (n = 5) and ulcer (n = 5). mRNA expression levels of CD123 were estimated quantitatively as described in the Methods section. Statistical significance of differences between groups was determined by Kruskal-Wallis tests.
(TIF)

S1 File Supporting information file with the individual relevant data of this study (anonymous). (XLSX)
