# Supplementary figures and images for: Myeloid dendritic cells stimulated by thymic stromal lymphopoietin promote Th2 immune responses and the pathogenesis of oral lichen planus
Source: PLoS One. 2017 Mar 9;12(3):e0173017. doi: 10.1371/journal.pone.0173017 (PMC5344337; doi:10.1371/journal.pone.0173017)

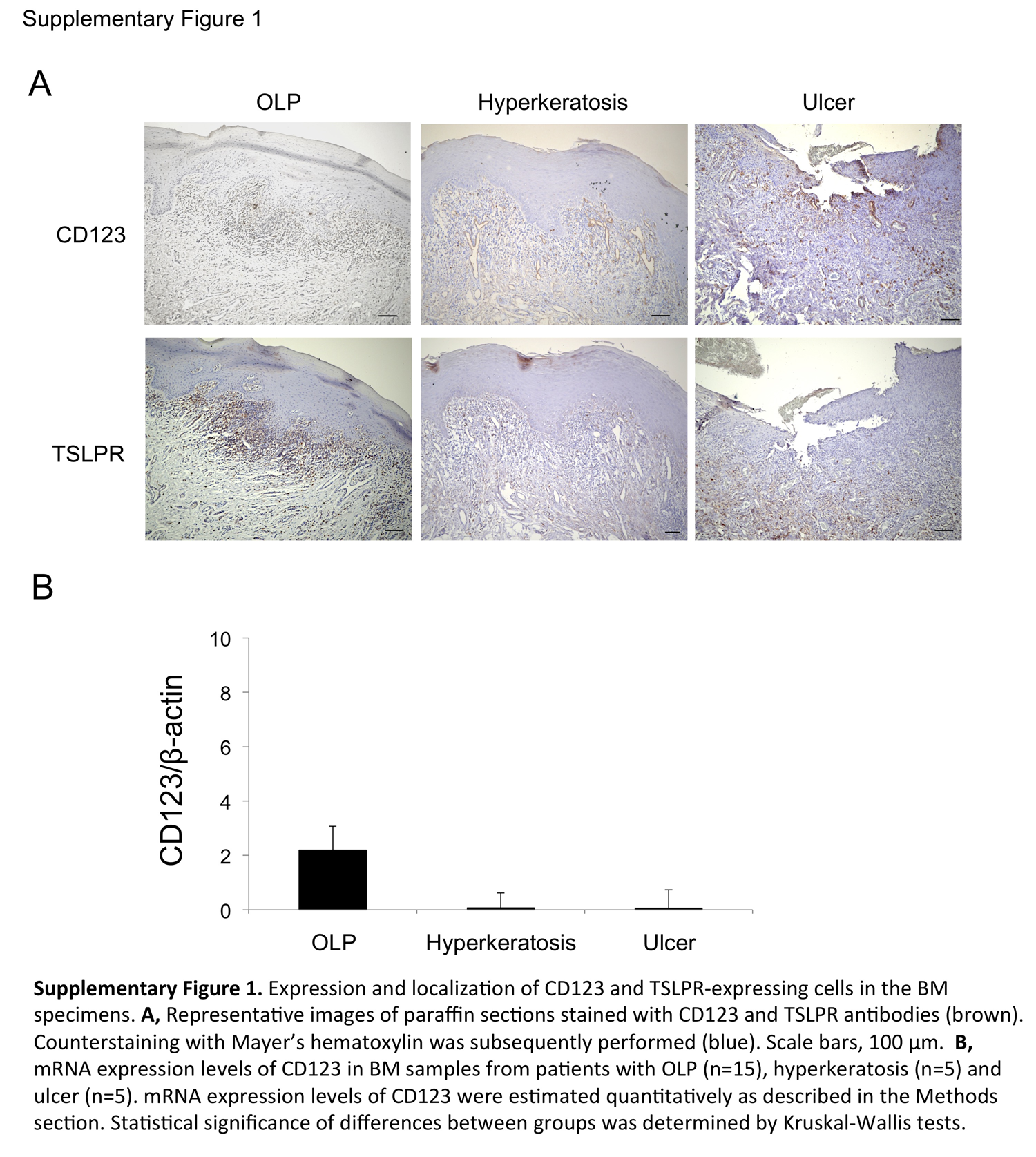

Supplement: S1 Fig — A, Representative images of paraffin sections stained with CD123 and TSLPR antibodies (brown). Counterstaining with Mayer’s hematoxylin was subsequently performed (blue). Scale bars, 100 μm. B, mRNA expression levels of CD123 in BM samples from patients with OLP (n = 15), hyperkeratosis (n = 5) and ulcer (n = 5). mRNA expression levels of CD123 were estimated quantitatively as described in the Methods section. Statistical significance of differences between groups was determined by Kruskal-Wallis tests. (TIF) [file pone.0173017.s001.tif]
